# Elevation of serum interleukins 8, 4, and 1β levels in patients with gastrointestinal low-grade B-cell lymphoma

**DOI:** 10.1038/srep18434

**Published:** 2015-12-17

**Authors:** Tomoko Miyata-Takata, Katsuyoshi Takata, Tomohiro Toji, Naoe Goto, Senji Kasahara, Takeshi Takahashi, Akira Tari, Mai Noujima-Harada, Takafumi Miyata, Yasuharu Sato, Tadashi Yoshino

**Affiliations:** 1 Department of Pathology, Okayama University Graduate School of Medicine, Dentistry and Pharmaceutical Sciences, Japan; 2 Department of Pathology, Okayama Medical Center, Japan; 3 Department of Hematology, Gihoku Kosei Hospital, Japan; 4 Department of Hematology, Gifu City Hospital, Japan; 5 Department of Gastroenterology, Hiroshima Red Cross Hospital and Atomic-bomb Survivors Hospital, Japan; 6 Department of Computer Science and Engineering, Fukuoka Institute of Technology, Japan

## Abstract

Proinflammatory cytokines that are produced by helper T cells (Th) regulate immune reactions, facilitate class switching of B cells, and prolong the lifespan of B and T cells. Eradication therapy using antibiotics is sometimes effective against gastrointestinal (GI) malignant lymphoma, suggesting that the tumor development or progression is affected by the inflammatory microenvironment. In the present study, serum samples from 148 patients with various subtypes of malignant lymphoma were tested for 11 proinflammatory Th1/Th2 cytokines. In the comparison by subtype or GI lesions, serum interleukin (IL)-8 (*P* = 6.7E−05), IL-4 (*P* = 7.5E−05), and IL-1β (*P* = 0.0043) levels showed significant differences among subtypes, being particularly elevated in follicular lymphomas (FL) and mucosa-associated lymphoid tissue (MALT) lymphomas. Serum IL-8 levels were elevated in GI-FL and MALT lymphomas, and serum IL-4 and IL-1 β levels were elevated in MALT lymphomas. These findings show that GI low-grade B-cell lymphoma could develop against the background of an inflammatory microenvironment. Thus, these cytokines may be useful as diagnostic markers and could provide new insights into tumor development.

Eradication therapy using antibiotics is often effective against gastrointestinal (GI) mucosa–associated lymphoid tissue (MALT) lymphoma. More than 90% of gastric MALT lymphoma cases are associated with *Helicobacter pylori* (HP) infection, and 75% of them regress after HP eradication^1^. In addition to MALT lymphoma, some cases of rectal MALT lymphoma and gastric diffuse large B-cell lymphoma (DLBCL) show regression as a result of HP eradication therapy^2^,^3^,^4^,^5^, suggesting that development or progression of GI lymphoma is affected by the host inflammatory microenvironment or immune reactions. MALT lymphoma is the most frequent subtype in the stomach, whereas follicular lymphoma (FL) is most common in the duodenum^6^,^7^. Studies have shown that in contrast to the nodal subtype, intestinal FL shares characteristics with MALT lymphoma such as clinicopathological features, immunoglobulin repertoire, and gene expression^7^. This observation suggests that an immune reaction to some stimuli may have role in the tumor development or progression of intestinal FL.

Proinflammatory cytokines produced by helper T cells (Th) regulate immune reactions, and it is known that they facilitate class switching of B cells and prolong the lifespan of B and T cells^8^. Among patients with HP-related gastritis, several Th1/Th2 cytokines elevations were reported^9^. As for malignant lymphoma, relationship with Th1/Th2 cytokine profile is not well elucidated. In the present study, we tested the hypothesis that the profiles of serum Th1/Th2 cytokines of various lymphoma subtypes differ according to subtypes. We then focused on GI low-grade B-cell lymphoma, particularly FL and MALT lymphoma.

## Results

### Serum interleukin (IL)-8, IL-4 and IL-1β levels were different depending on a lymphoma subtype

Among the 11 analyzed cytokines, IL-8, IL-4, and IL-1β levels were significantly different from control depending on the lymphoma subtype using one-way analysis of variance among control and lymphoma subtypes (*P* = 6.69E−05, 7.46E−05, and 0.0043, respectively; Table 1). These three cytokines were also significantly different among the lymphoma subtypes (*P* = 1.63E−04, 1.15E−04, and 0.0040, respectively; Table 1). When we limited the analysis to three major subtypes (DLBCL, FL, and MALT lymphoma) and subdivided the cases by the presence or absence of GI lesions, these three cytokines also showed serum levels significantly different from controls using one-way analysis of variance among control and lymphoma subtypes (*P* = 0.0039, 0.00019, and 0.024, respectively; Table 2). These three cytokines were also significantly different among the lymphoma subtypes (*P* = 0.021, 0.00076, and 0.045, respectively; Table 2).

### Serum IL-8 level was elevated in GI low-grade B-cell lymphoma

**Figure 1:**
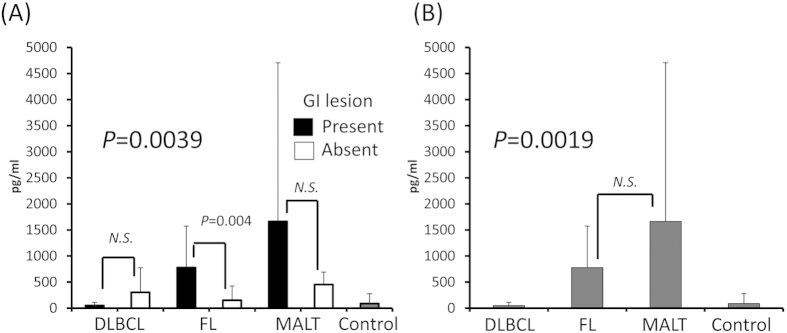
Serum IL-8 level according to lymphoma subtype and with/without GI lesions. (**A**) Serum IL-8 level was elevated in GI FL and MALT lymphomas. The IL-8 level was elevated in FL with GI lesions, but not in MALT lymphomas. (**B**) Among cases with GI lesions, serum IL-8 was elevated in FL and MALT lymphomas. There was no difference between FL and MALT lymphomas with GI lesions. IL: interleukin, GI: gastrointestinal, DLBCL: diffuse large B-cell lymphoma, FL: follicular lymphoma, MALT: mucosa-associated lymphoid tissue.

The serum IL-8 level was elevated in FL and MALT lymphomas with GI lesions (*P* = 0.0039; Fig. 1A,B), and there was no difference between these two subtypes with GI lesions (*P* = 0.25; Fig. 1A). In FL, the serum IL-8 level in cases with GI lesions was higher than that in cases without GI lesions, but in MALT lymphomas, there was no difference between cases with and without GI lesions (Fig. 1A). Although the IL-8 value was higher in GI(−)DLBCL than in GI(+)DLBCL, it was not significantly different (*P* = 0.138).

**Figure 2:**
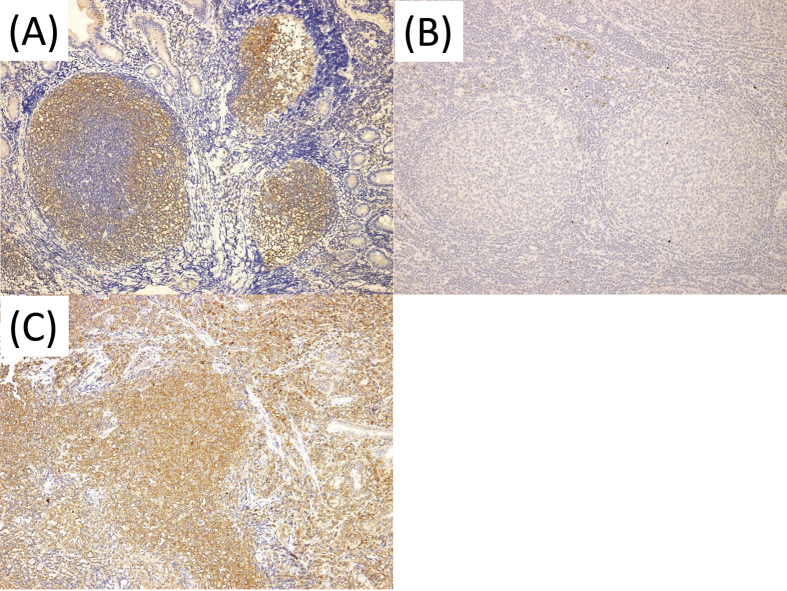
Immunohistochemical analysis of IL-8RB in representative FL and MALT lymphoma cases. Intestinal FL (**A**) and gastric MALT lymphoma (**C**) tested positive for IL-8RB, but nodal FL tested negative (**B**). IL-8RB: interleukin-8 receptor β, GI: gastrointestinal, FL: follicular lymphoma, MALT: mucosa-associated lymphoid tissue.

As for the 17 GI-FL, 16 nodal FL, and 10 GI-MALT lymphoma cases, interleukin-8 receptor β (IL-8RB) (CXCR2) protein expression was assessed using immunohistochemical analysis. Among the FL cases, IL-8RB expression was frequently observed in GI-FL cases (GI-FL: 17/17, nodal FL: 8/16; *P* = 0.0093; Fig. 2A,B), whereas all MALT lymphoma cases showed expression of IL-8RB (Fig. 2C). This result meant that the receptor of IL-8 was expressed in tumor tissue.

### Serum IL-4 and IL-1β levels were elevated in GI-MALT lymphoma cases

**Figure 3:**
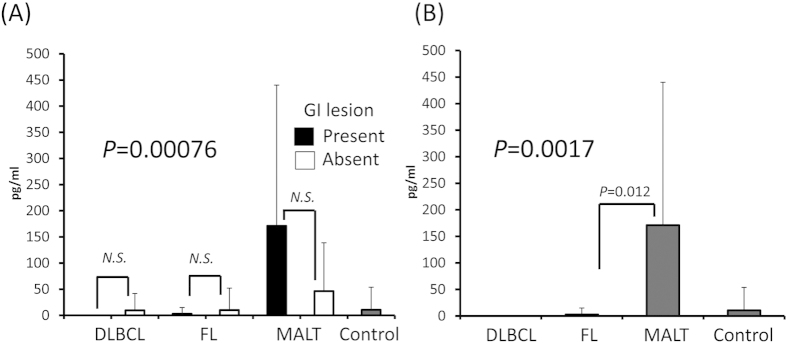
IL-4 serum levels by lymphoma subtypes and with/without GI lesions. (**A**) Serum IL-4 levels were elevated in MALT lymphomas regardless of GI lesions. There were no differences between MALT lymphomas with and without GI lesions. (**B**) Among cases with GI lesions, serum IL-4 level was elevated in MALT lymphomas. IL: interleukin, GI: gastrointestinal, DLBCL: diffuse large B-cell lymphoma, FL: follicular lymphoma, MALT: mucosa-associated lymphoid tissue.

**Figure 4:**
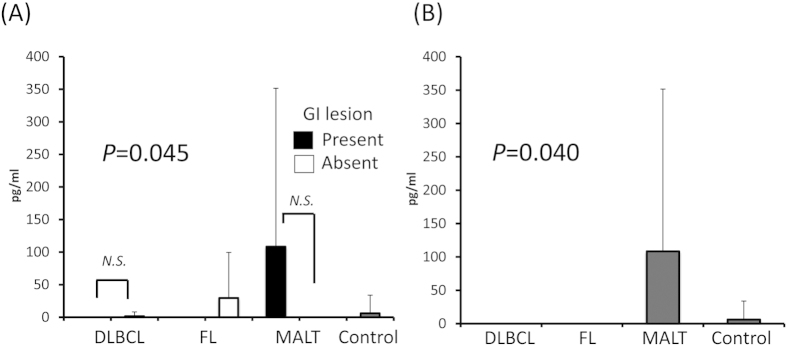
Serum IL-1β levels by lymphoma subtypes and with/without GI lesions. (**A**) Serum IL-4 levels were elevated in MALT lymphomas regardless of GI lesions. There was no significant difference between MALT lymphomas with and without GI lesions. (**B**) Among cases with GI lesions, the serum IL-1β level was elevated in MALT lymphomas. IL: interleukin, GI: gastrointestinal, DLBCL: diffuse large B-cell lymphoma, FL: follicular lymphoma, MALT: mucosa-associated lymphoid tissue.

Serum IL-4 and IL-1β levels were elevated in MALT lymphoma cases compared with healthy controls (*P* = 0.00019 and 0.024, respectively; Figs 3A and 4A). Both cytokines were also elevated when we analyzed only serum samples from MALT lymphoma cases with GI lesions (Figs 3B and 4B). In MALT lymphoma cases, the serum levels of both cytokines were not linked to the presence of GI lesions (*P* = 0.39 and 0.40, respectively; Figs 3A and 4A). Similarly, IL-4 and IL-1β were higher in GI−DLBCL than in GI+DLBCL, but they were not significantly different (*P* = 0.410, 0.045, respectively).

## Discussion

IL-8 (CXCL8) was first identified as a neutrophil chemoattractant that belongs to the CXC chemokine family^10^. IL-8 is a proinflammatory chemokine and an angiogenic factor that mediates various inflammatory responses and is associated with tumor progression^11^. In non–small cell lung carcinoma, IL-8 is overexpressed under the influence of KRAS and enhances the stromal response by inducing inflammation and angiogenesis, which are associated with tumor proliferation and negatively correlate with patients’ survival^12^.

After antigen stimulation in the Peyer’s patch, B cells undergo class switching to IgA expressing α4β7 integrin and then home to the intestine via MAdCAM-1, which is expressed on endothelial cells of intestinal blood vessels^13^. Judging by the gene expression analysis reported previously, MAdCAM-1 and CCL20 were overexpressed in FL and MALT lymphoma tissue of the GI tract compared with the nodal counterpart^14^. This overexpression of MAdCAM-1 may be caused by the IL-8–induced angiogenesis.

IL-4 is produced by Th2 cells, mast cells, and basophils and activates the JAK/STAT and PI3K pathways. In addition to the association of IL-4 with inflammatory responses and allergic reactions, B cells express IL-4 receptor, which promotes their proliferation as a co-mitogen, controls class switching to IgE or IgG4, and prolongs the cellular lifespan. IL-4 also makes B cells express THY1 by associating with lipopolysaccharides^8^,^15^. IL-1β is a component of the IL-1 complex and is linked to several inflammatory processes by activating macrophages. In addition to IL-8, we examined IL-4 receptor α (IL-4RA) immunostaining on 30 cases (10 GI FL, 10 nodal FL, and 10 GI MALT). In contrast to IL-8RB, IL-4RA was weakly positive for more than half of the cases regardless of the subtypes (*P* = 0.63) and was not reflected high serum IL-4 value in MALT lymphoma. (data not shown) It is suggested that IL-4 acts indirectly on tumor cells. Unfortunately an adequate level of IL-1 receptor antibody was not available.

HP is known to be a major factor in the MALT lymphoma development. According to some researchers who performed cytokine expression analysis of HP-related and HP-unrelated gastritis, several Th1/Th2 cytokine combinations participate in the HP-related inflammation^9^. Elevation of IL-4, IL-8, IL-1β, IL-6, IL-10, tumor necrosis factor (TNF)-α, and interferon (IFN)-γ levels was also observed. In HP-related gastritis caused by the CagA-positive strain, elevation of IL-1β and IL-18 levels was reported^9^.

In the present study, elevation of IL-4, IL-1β, and IL-8 levels was detected in MALT lymphomas. This change may be caused by the HP-related inflammation. In another report, IL-4 production was found to be increased when a patient’s peripheral blood mononuclear cells (PBMCs) were stimulated by HP hsp60 or HP lysate^16^. Gene pleomorphism of the IL-4 gene and IL-1 receptor antagonist (IL-1RN) gene was reported to negatively correlate with HP-related gastric cancer^17^, suggesting that reactivity to such cytokines is linked to tumor development. In the present study, the correlation of HP infection with changes in cytokine production was not observed probably because of the insufficient sample size (Supplementary Figure).

When confined to low-grade B-cell lymphoma, most of the cases were primary GI cases and a few cases were those of secondary GI involvement in this study. We could not examine if there were any clinicopathological differences between the primary and secondary cases because the number of secondary cases was a few. However, it is suggested that the cytokine profiles shown in this study reflected those of primary GI low-grade B-cell lymphoma.

Although the main lesion of primary GI FL is often in the duodenum, total GI tract screening using double-balloon endoscopy revealed that 85% of the cases had lymphoma lesions that were broadly located in the GI tract^7^. In addition, some cases of MALT lymphoma of the stomach have concomitant lesions in the duodenum or colon. In our series, there was small number of patients whose GI lesion restricted to single organ were 6 cases (all stomach cases) for MALT, 13 (duodenum: 10, ileum: 3) for FL, 7 (stomach: 5, ileum: 1, colon: 1) for DLBCL. Considering these facts, HP +/− gastritis patients were not necessarily fit as adequate controls for this study as well as the inflammatory disease patients affecting corresponding organs, such as duodenitis, ileitis and colitis, because comparison among other subtypes, would be difficult. Therefore, we included healthy subjects as the controls in this study.

It was reported that the clinical behavior of the GI FL cases was very indolent, similar to MALT lymphoma but in contrast to that of the nodal type^7^,^18^. Moreover GI FL was reported to have similar characteristics with MALT lymphoma, both in the immunohistochemical and molecular aspects including gene expression profiles^14^,^19^. In the present study, GI FL and MALT lymphoma had similar elevations in serum IL-8; to discriminate MALT from GI FL, IL-4 and 1β elevations could be used. In this study, the functions of the cytokines indicated for the specific lymphoma subtypes were not investigated and warrant further study. For example, lymphoma development under the influence of specific cytokines using transgenic mice holding t(14;18) needs to be examined. To our knowledge, there is no report analyzing the proinflammatory cytokine profiles that are specific for GI lymphoma, and we believe that the relevance of this study is in the discovery of important serum cytokines, which could be the factors involved in the pathogenesis of lymphoma.

In summary, analysis of Th1/Th2 cytokine levels in serum samples shows that IL-8 levels were elevated in GI FL and MALT lymphomas, whereas IL-4 and IL-1β levels were elevated in MALT lymphomas. These results show that GI low-grade B-cell lymphoma probably develops in an inflammatory microenvironment. This finding needs confirmation with a larger sample size, particularly for the utility of these cytokines as auxiliary diagnostic biomarkers. Further studies of the mechanisms involving these cytokines in the tumor microenvironment would be warranted and may lead to new therapeutic targets.

## Methods

### Clinical samples

Serum samples from 148 patients with malignant lymphoma were collected at our collaborating hospitals: Gifu University Hospital (June 2004–March 2012), Gifu City Hospital (March 2010–April 2015), and Hiroshima Red Cross Hospital and Atomic-bomb Survivors Hospital (June 2011–May 2012). The samples from the patients included in this study are described in Table 3. The patient age had a median age of 62 years (range 41–83 years), and the male to female ratio was 16:15. Serum samples for all cases, except three, were taken before treatment. The three cases were as follows: 1 mantle cell lymphoma (MCL), 1 Hodgkin lymphoma (HL), and 1 MALT lymphoma (HP eradication therapy alone). The other clinical characteristics were obtained from 31 cases. The numbers of serum samples corresponding to each lymphoma subtype were as follows: 55 cases of DLBCLs including 2 intravascular large B-cell lymphomas (IVL), 36 FLs, 15 MALT lymphomas, and 42 other lymphomas (2 angioimmunoblastic T-cell lymphomas, 2 adult T-cell leukemias/ lymphomas, 2 acute lymphoblastic leukemias, 2 non-HLs, 2 Burkitt lymphomas, 1 hairy cell leukemia, 1 hepatosplenic T-cell lymphoma, 5 HLs, 3 MCLs, 4 natural killer (NK)/T-cell lymphomas, 15 peripheral T-cell lymphomas, 2 small lymphocytic lymphomas, and 1 cutaneous follicle center lymphoma). Serum samples from 21 healthy volunteers served as controls. Approval for the study protocol was obtained from the institutional review board at Okayama University Graduate School of Medicine, Dentistry, and Pharmaceutical Sciences and similar boards at the collaborating hospitals. Informed consent was obtained from all subjects. All study procedures were conducted in accordance with the guidelines of the Declaration of Helsinki. In our hospital and other collaborating centers, diagnosis was based on the current WHO classification using appropriate immunostaining, such as CD20, 10, 5, BCL2, and cyclinD1 for low-grade B-cell lymphoma, and CD20, 10, and 5 for DLBCL^6^. In the analysis comparing the presence of GI lesions in DLBCL, FL, and MALT, cases of IVL (n = 2) and composite MALT and DLBCL (n = 1) were excluded. The behavior of composite MALT and DLBCL could not be investigated with a low sample number. Relevant information concerning involved sites was obtained from 81 out of 106 cases of major 3 subtypes (DLBCL, FL, and MALT). Among them, 37 had GI lesions and 28 of these were primary sites of lymphoma (case numbers of “primary GI cases” and “cases with secondary involvement or suspicious for secondary involvement of the GI tract” were 1 and 7 for DLBCL, 17 and 1 for FL, and 10 and 1 for MALT lymphoma, respectively).

### Cytokine analysis

Eleven kinds of cytokines [IFN-γ, IL-1β, IL-2, IL-4, IL-5, IL-6, IL-8, IL-10, IL-12p70, TNF-α, and TNF-β] were analyzed using the FlowCytomix Human Th1/Th2 11plex Kit BMS810FF (eBioscience, Vienna, Austria) and MACSQuant Analyzer (Miltenyi Biotec, Bergisch Gladbach, Germany).

### Immunohistochemical analysis

Biopsy samples were fixed in 10% buffered formalin embedded in paraffin blocks and subjected to immunohistochemical analysis as described previously^20^. The clone of the antibody and dilution were 48311 and 1:100 for IL-8RB, and 25463 and 1:500 for IL-4RA, respectively (R&D Systems, Inc., Minneapolis, USA).

### Statistical analysis

One-way analysis of variance was used. For statistical tests *P* < 0.05 was set as significant.

## Additional Information

**How to cite this article**: Miyata-Takata, T. *et al.* Elevation of serum interleukins 8, 4, and 1β levels in patients with gastrointestinal low-grade B-cell lymphoma. *Sci. Rep.* **5**, 18434; doi: 10.1038/srep18434 (2015).

## Supplementary Material

Supplementary Figure

## Figures and Tables

**Table 1:** Cytokine values according to each histological subtype.

	Subtype (n)	DLBCL (55)	FL (36)	MALT(15)	Others (42)	(pg/ml)	P*	P**
						Normal (21)		
IL8	Median	180.193	462.404	1340.327	159.927	88.731	**6.69E-05**	**1.63E-04**
	SD	341.955	667.029	2634.167	287.289	188.895		
IL4	Median	18.919	6.189	137.726	26.983	10.565	**7.46E-05**	**1.15E-04**
	SD	56.707	32.751	238.447	69.04	43.259		
IL1β	Median	2.906	13.856	79.461	7.408	6.061	**0.0043**	**0.0040**
	SD	17.617	49.704	211.445	24.653	27.775		
IL12p70	Median	26.78	0.753	11.067	120.256	26.915	0.65	0.54
	SD	185.671	4.52	31.793	715.009	87.175		
IFNγ	Median	22.305	0	7.679	217.567	0	0.076	0.07
	SD	99.65	0	24.516	787.469	0		
IL2	Median	30.463	29.98	54.789	28.705	29.6	0.97	0.92
	SD	168.096	64.583	142.198	117.611	135.642		
IL10	Median	814.164	1.696	13.149	105.894	2.161	0.31	0.28
	SD	3733.864	4.765	23.435	401.716	9.905		
IL6	Median	31.509	7.834	21.831	17.632	1.169	0.73	0.77
	SD	155.883	29.162	75.664	68.259	5.357		
IL5	Median	16.509	1.856	11.153	13.955	1.021	0.82	0.81
	SD	104.195	10.185	43.197	57.841	4.679		
TNFα	Median	2.117	19.336	32.285	19.269	3.104	0.14	0.15
	SD	11.309	78.945	71.478	50.049	14.226		
TNFβ	Median	0	2.875	0	5.419	14.509	0.39	0.54
	SD	0	17.252	0	32.034	66.487		

DLBCL: diffuse large B-cell lymphoma, FL: follicular lymphoma, MALT: mucosa-associated lymphoid tissue lymphoma

*P values were calculated among lymphoma subtypes and normal controls.

**P values were calculated among lymphoma subtypes.

**Table 2:** Cytokine values of each lymphoma subtype according to presence or absence of gastrointestinal lesion.

Subtype		DLBCL		FL		MALT		(pg/ml)	P*	P**
GI lesion (n)		Present (8)	Absent (23)	Present (18)	Absent (17)	Present (11)	Absent (4)	Normal (21)		
IL8	Median	48.626	304.683	777.218	150.861	1662.842	453.41	88.731	**0.0039**	**0.021**
	SD	62.874	469.097	797.085	273.918	3044.377	238.565	188.895		
IL4	Median	0	9.638	2.871	10.194	170.979	46.28	10.565	**0.00019**	**0.00076**
	SD	0	32.315	12.179	42.031	269.2	92.56	43.259		
IL1β	Median	0	1.422	0	29.342	108.355	0	6.061	**0.024**	**0.045**
	SD	0	6.821	0	70.153	243.205	0	27.775		
IL12p70	Median	5.565	59.874	1.507	0	15.092	0	26.915	0.86	0.823
	SD	15.74	287.145	6.392	0	36.72	0	87.175		
IFNγ	Median	0	43.767	0	0	10.471	0	0	0.41	0.502
	SD	0	150.935	0	0	28.448	0	0		
IL2	Median	0	64.992	39.635	22.088	74.713	0	29.6	0.89	0.841
	SD	0	258.524	73.37	57.087	163.312	0	135.642		
IL10	Median	236.965	1453.79	0.149	3.433	15.176	7.573	2.161	0.56	0.619
	SD	529.503	5665.383	0.634	6.57	26.136	15.145	9.905		
IL6	Median	0	21.623	6.078	10.155	29.176	1.633	1.169	0.53	0.699
	SD	0	59.4	9.903	41.751	88.258	3.265	5.357		
IL5	Median	0.254	39.062	3.389	0.343	15.209	0	1.021	0.68	0.735
	SD	0.718	160.41	14.378	1.414	50.443	0	4.679		
TNFα	Median	0	0.776	25.784	13.647	38.192	16.04	3.104	0.49	0.583
	SD	0	3.722	109.392	28.535	81.853	32.08	14.226		
TNFβ	Median	0	0	5.751	0	0	0	14.509	0.77	0.635
	SD	0	0	24.398	0	0	0	66.487		

DLBCL: diffuse large B-cell lymphoma, FL: follicular lymphoma, MALT: mucosa-associated lymphoid tissue lymphoma, GI: gastrointestinal

*P values were calculated among lymphoma subtypes and normal controls.

**P values were calculated among lymphoma subtypes.

**Table 3:** Cases included in this study.

Histology	Total	Gastrointestinal lesion	
		Present	Absent
DLBCL	55	8	23
FL	36	18	17
MALT	15	11	4
Others	42		
Normal	21		

DLBCL: diffuse large B-cell lymphoma, FL: follicular lymphoma,

MALT: mucosa-associated lymphoid tissue lymphoma.

## References

[b1] Wotherspoon A. C. *et al.* Regression of primary low-grade B-cell gastric lymphoma of mucosa-associated lymphoid tissue type after eradication of Helicobacter pylori. Lancet 342, 575–577 (1993). 8102719 10.1016/0140-6736(93)91409-f

[b2] Nosaka K. *et al.* Regression of primary rectal MALT lymphoma after Helicobacter pylori eradication. Rinsho Ketsueki 55, 948–952 (2014). 25186484

[b3] Tari A. *et al.* Predictive value of endoscopy and endoscopic ultrasonography for regression of gastric diffuse large B-cell lymphomas after Helicobacter pylori eradication. Dig Endosc 21, 219–227, 10.1111/j.1443-1661.2009.00896.x (2009). 19961519

[b4] Ng W. W. *et al.* Regression of high-grade gastric mucosa-associated lymphoid tissue lymphoma with Helicobacter pylori after triple antibiotic therapy. Gastrointestinal endoscopy 51, 93–96 (2000). 10625811 10.1016/s0016-5107(00)70399-3

[b5] Kuo S. H. *et al.* Helicobacter pylori eradication therapy is effective in the treatment of early-stage H pylori-positive gastric diffuse large B-cell lymphomas. Blood 119, 4838–4844; quiz 5057, 10.1182/blood-2012-01-404194 (2012). 22403257

[b6] Swerdlow S. H. *et al.* eds. WHO Classification of Tumours of Haematopoietic and Lymphoid Tissues. 4th edn, 281–284 (2008).

[b7] Takata K. *et al.* Primary gastrointestinal follicular lymphoma involving the duodenal second portion is a distinct entity: a multicenter, retrospective analysis in Japan. Cancer Sci 102, 1532–1536, 10.1111/j.1349-7006.2011.01980.x (2011). 21561531

[b8] Nelms K., Keegan A. D., Zamorano J., Ryan J. J. & Paul W. E. The IL-4 receptor: signaling mechanisms and biologic functions. Annu Rev Immunol 17, 701–738, 10.1146/annurev.immunol.17.1.701 (1999). 10358772

[b9] Goll R. *et al.* Helicobacter pylori stimulates a mixed adaptive immune response with a strong T-regulatory component in human gastric mucosa. Helicobacter 12, 185–192, 10.1111/j.1523-5378.2007.00495.x (2007). 17492997

[b10] Yoshimura T. *et al.* Purification of a human monocyte-derived neutrophil chemotactic factor that has peptide sequence similarity to other host defense cytokines. Proc Natl Acad Sci USA 84, 9233–9237 (1987). 3480540 10.1073/pnas.84.24.9233 PMC299727

[b11] Waugh D. J. & Wilson C. The interleukin-8 pathway in cancer. Clin Cancer Res 14, 6735–6741, 10.1158/1078-0432.ccr-07-4843 (2008). 18980965

[b12] Sunaga N. *et al.* Clinicopathological and prognostic significance of interleukin-8 expression and its relationship to KRAS mutation in lung adenocarcinoma. Br J Cancer 110, 2047–2053, 10.1038/bjc.2014.110 (2014). 24577055 PMC3992490

[b13] Berlin C. *et al.* Alpha 4 beta 7 integrin mediates lymphocyte binding to the mucosal vascular addressin MAdCAM-1. Cell 74, 185–195 (1993). 7687523 10.1016/0092-8674(93)90305-a

[b14] Takata K. *et al.* Duodenal follicular lymphoma: comprehensive gene expression analysis with insights into pathogenesis. Cancer Sci 105, 608–615, 10.1111/cas.12392 (2014). 24602001 PMC4317842

[b15] Kelly-Welch A. E., Hanson E. M., Boothby M. R. & Keegan A. D. Interleukin-4 and interleukin-13 signaling connections maps. Science 300, 1527–1528, 10.1126/science.1085458 (2003). 12791978

[b16] Yamasaki R. *et al.* Immune response in Helicobacter pylori-induced low-grade gastric-mucosa-associated lymphoid tissue (MALT) lymphoma. J Med Microbiol 53, 21–29 (2004). 14663101 10.1099/jmm.0.05348-0

[b17] Seno H. *et al.* Novel interleukin-4 and interleukin-1 receptor antagonist gene variations associated with non-cardia gastric cancer in Japan: comprehensive analysis of 207 polymorphisms of 11 cytokine genes. J Gastroenterol Hepatol 22, 729–737, 10.1111/j.1440-1746.2007.04934.x (2007). 17444864

[b18] Schmatz A. I. *et al.* Primary follicular lymphoma of the duodenum is a distinct mucosal/submucosal variant of follicular lymphoma: a retrospective study of 63 cases. J Clin Oncol 29, 1445–1451, 10.1200/jco.2010.32.9193 (2011). 21383289

[b19] Takata K. *et al.* Duodenal and nodal follicular lymphomas are distinct: the former lacks activation-induced cytidine deaminase and follicular dendritic cells despite ongoing somatic hypermutations. Mod Pathol 22, 940–949, 10.1038/modpathol.2009.51 (2009). 19396151

[b20] Takata K. *et al.* Duodenal follicular lymphoma lacks AID but expresses BACH2 and has memory B-cell characteristics. Mod Pathol 26, 22–31, 10.1038/modpathol.2012.127 (2013). 22899287

