# Supplementary material for: Elevation of serum interleukins 8, 4, and 1β levels in patients with gastrointestinal low-grade B-cell lymphoma
Source: Sci Rep. 2015 Dec 17;5:18434. doi: 10.1038/srep18434 (PMC4682061; doi:10.1038/srep18434)

**Elevation of serum interleukins 8, 4, and 1β levels in patients with gastrointestinal low-grade B-cell lymphoma**

Tomoko Miyata-Takata^1#^, Katsuyoshi Takata^1#*^, Tomohiro Toji^1,2#^, Naoe Goto^3^,

Senji Kasahara^4^, Takeshi Takahashi^4^, Akira Tari^5^, Mai Noujima-Harada^1^, Takafumi Miyata^6^, Yasuharu Sato^1^, Tadashi Yoshino^1^.

Affiliations:

^1^Department of Pathology, Okayama University Graduate School of Medicine, Dentistry and Pharmaceutical Sciences, Japan

^2^Department of Pathology, Okayama Medical Center, Japan

^3^Department of Hematology, Gihoku Kosei Hospital, Japan

^4^Department of Hematology, Gifu City Hospital, Japan

^5^Department of Gastroenterology, Hiroshima Red Cross Hospital and Atomic-bomb Survivors Hospital, Japan

^6^Department of Computer Science and Engineering, Fukuoka Institute of Technology, Japan

# These authors are equally contributed.

*Corresponding author:

Katsuyoshi Takata, M.D., Ph.D.

Department of Pathology, Okayama University Graduate School of Medicine, Dentistry and Pharmaceutical Sciences

2-5-1 Shikata-cho, kita-ku, Okayama City, Okayama 700-8558, Japan

Tel: (+81)-86-235-7150

Fax: (+81)-86-235-7156

E-mail: katsuyoshi.t@h5.dion.ne.jp


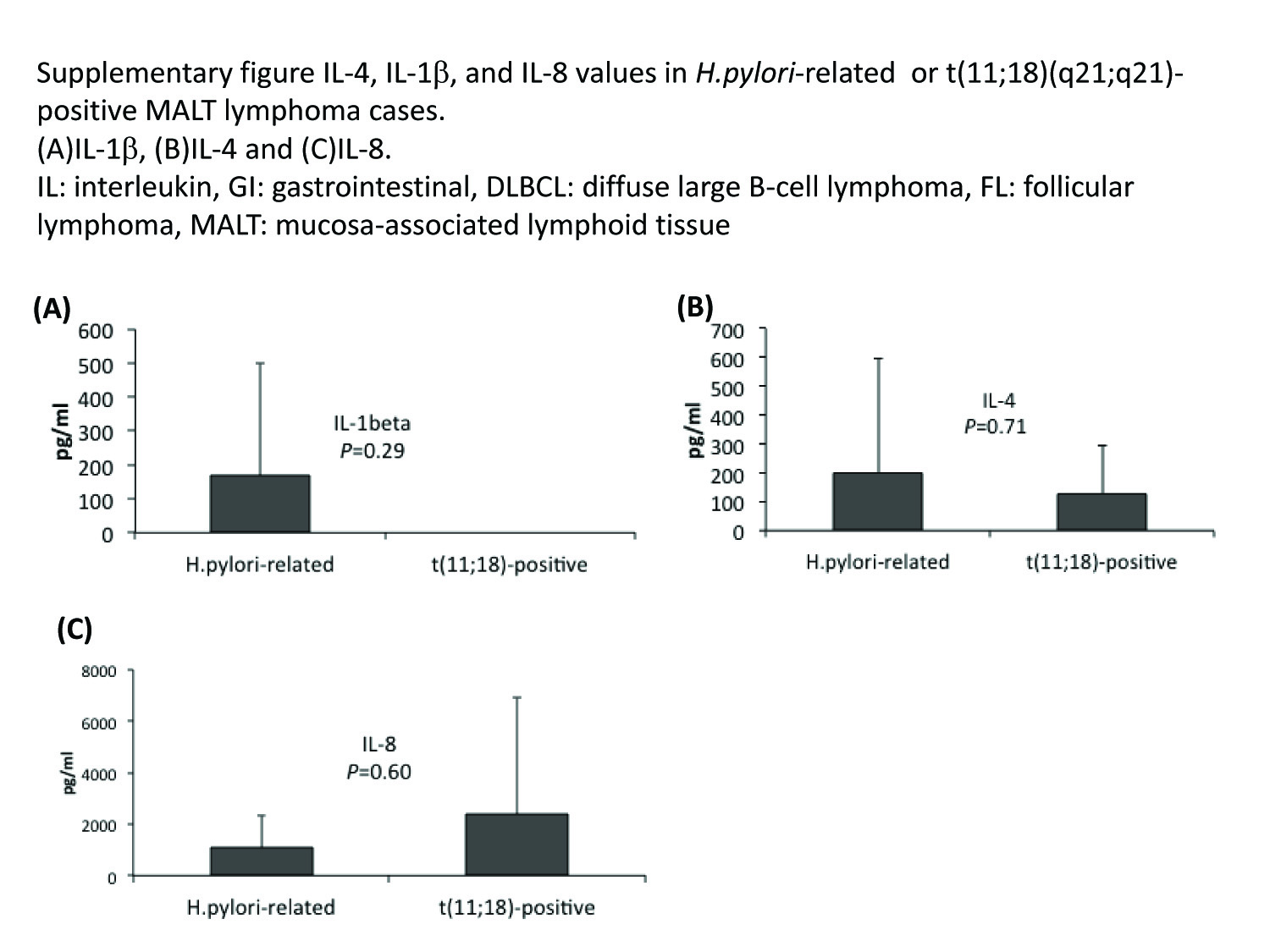

Supplement: Supplementary Figure [file srep18434-s1.doc]
